# Development, reliability and validity of infectious disease specialist Nurse’s Core competence scale

**DOI:** 10.1186/s12912-021-00757-2

**Published:** 2021-11-17

**Authors:** Chao Wu, Jiaran Yan, Jing Wu, Ping Wu, Feixia Cheng, Lina Du, Yanling Du, Shang Lei, Hongjuan Lang

**Affiliations:** 1 Nursing Department, Fourth Military Medical University, No.169 Changle West Road, Xi’an, 710032 Shaanxi China; 2 Tangdu Hospital of Air Force Military Medical University, Shaanxi, China; 3 Tongji Hospital of Huazhong University of Science and Technology, Hubei, China; 4 Naval University of Engineering, Hubei, China; 5 986th Hospital of Air Force Military Medical University, Shaanxi, China; 6 Department of Health Statistics, Fourth Military Medical University, No.169 Changle West Road, Xi’an, 710032 Shaanxi China

**Keywords:** Infectious disease specialist nurse, Core competence, Questionnaire development, Reliability, Validity, Exploratory factor analysis, Confirmatory factor analysis

## Abstract

**Aim:**

This study aims to develop an instrument to measure infectious disease specialist nurses’ core competence and examining the scale’s validity and reliability.

**Background:**

With the increase of infectious diseases, more and more attention has been paid to infectious disease nursing care. The core competence of the infectious disease specialist nurses is directly related to the quality of nursing work. In previous researches, infectious disease specialist nurses’ core competence was measured by the tools developed for general nurses instead of specialized tools, which made it difficult to clarify the core competence of nurses in infectious diseases department.

**Methods:**

Preliminary items were developed through literature review, theoretical research, qualitative interview and Delphi method. The confirmed 47 items were applied in the two rounds of data collection. Evaluation data on 516 infectious disease specialist nurses’ core competence in the first round were utilized to preliminarily evaluate and explore the scale’s constrution, while evaluation data on 497 infectious disease specialist nurses’ core competence in the second round were utilized to do reliability analysis and validity analysis. In this study, factor analysis, Cronbach’s α, Pearson correlation coefficients were all adopted.

**Results:**

The final scale is composed of 34 items and 5 factors, and adopted the 5-point scoring method. The factors are Professional Development Abilities, Infection Prevention and Control Abilities, Nursing Abilities for Infectious Diseases, Professionalism and Humanistic Accomplishment, and Responsiveness to Emergency Infectious Diseases. The explanatory variance of the five factors was 75.569%. The reliability and validity of the scale is well validated. The internal consistency, split-half reliability and test-retest reliability were 0.806, 0.966 and 0.831 respectively. The scale has good structural validity and content validity. The content validity was 0.869. Discrimination analysis showed that there were significant differences in the scores of core competence and its five dimensions among infectious disease specialist nurses of different ages, working years in infectious diseases, titles, educational background, marital status and wages (all *P* < 0.05).

**Conclusions:**

The proposed scale takes on high reliability and validity, and is suitable for assessing the infectious disease specialist nurses’ core competence.

**Relevance to clinical practice:**

This scale provides a reference for clinical assessment of infectious disease nursing.

## Contribution of the paper

**What is already known?**

- With the spread of infectious diseases all over the world, the importance of specialized nurses for infectious diseases has become increasingly prominent.
- The existing researches have scales for the evaluation of the core competence of general nurses and other specialist nurses.

**What this paper adds**

- This study clarified the ability that infectious disease specialist nurses should have.
- The Infectious Disease Specialist Nurses Core Competence Scale was developed, with good reliability and validity. It provides a reference for clinical assessment of infectious disease nursing.
- Through discrimination analysis, our study preliminarily explored the influencing factors of the core competence of infectious disease specialist nurses, including ages, working years, titles, educational background, marital status and wages

## Introduction

In recent years, the global epidemic caused by infectious diseases emerged continuously [1, 2]. The epidemic situation of infectious diseases such as yellow fever, Ebola hemorrhagic fever and Dengue fever is very severe around the world [3–5]. In particular, the COVID-19 which broke out at the end of 2019 had spread worldwide in a very short period of time, and human beings are still fighting against it until now [6, 7]. The infectious diseases not only pose great threats to human health, but also cause social panic within a certain range and affect economic and political stability [8].

The World Health Organization puts forward that under current situation, the public health work is facing huge challenges. Thus, nurses are playing more and more important roles and the requirements for professional nursing care is higher and higher [9]. Cultivating professional nursing talents has already becoming an important direction for nursing development in the new era (Mueller, Burggraf, & Crogan, 2020). That’s why infectious disease specialist nurses come into being. Their core competence is not only related to the quality of the infectious disease care, but also related to the effectiveness of the infectious disease treatment. It is of great significance to the protection of public health, economic development and social stability [10, 11].

The core competence of the nurses refers to the sum of knowledge, skills and comprehensive qualities required in the clinical nursing care (Chan, Lockhart, Schreiber, & Kronk, 2020). Through targeted measurement and evaluation of the specialist nurse’s core competence, it can be reference for their professional development, training, assessment and etc. At present, there are evaluation tools on core competence of the emergency nurses [12], operating theatre specialist nurses [13], gerontological specialist nurses [14] and general nurses [15]. But there is still a lack of quantifiable assessment tools for evaluating the infectious disease nurses’ core competence. Therefore, this study compiled the Infectious Disease Specialist Nurse’s Core Competence Scale, in hope that it can provide reference to the evaluation and assessment of the infectious disease specialist nurses so as to better improve the quality of the infectious disease nursing care.

## Methods

**Fig. 1:**
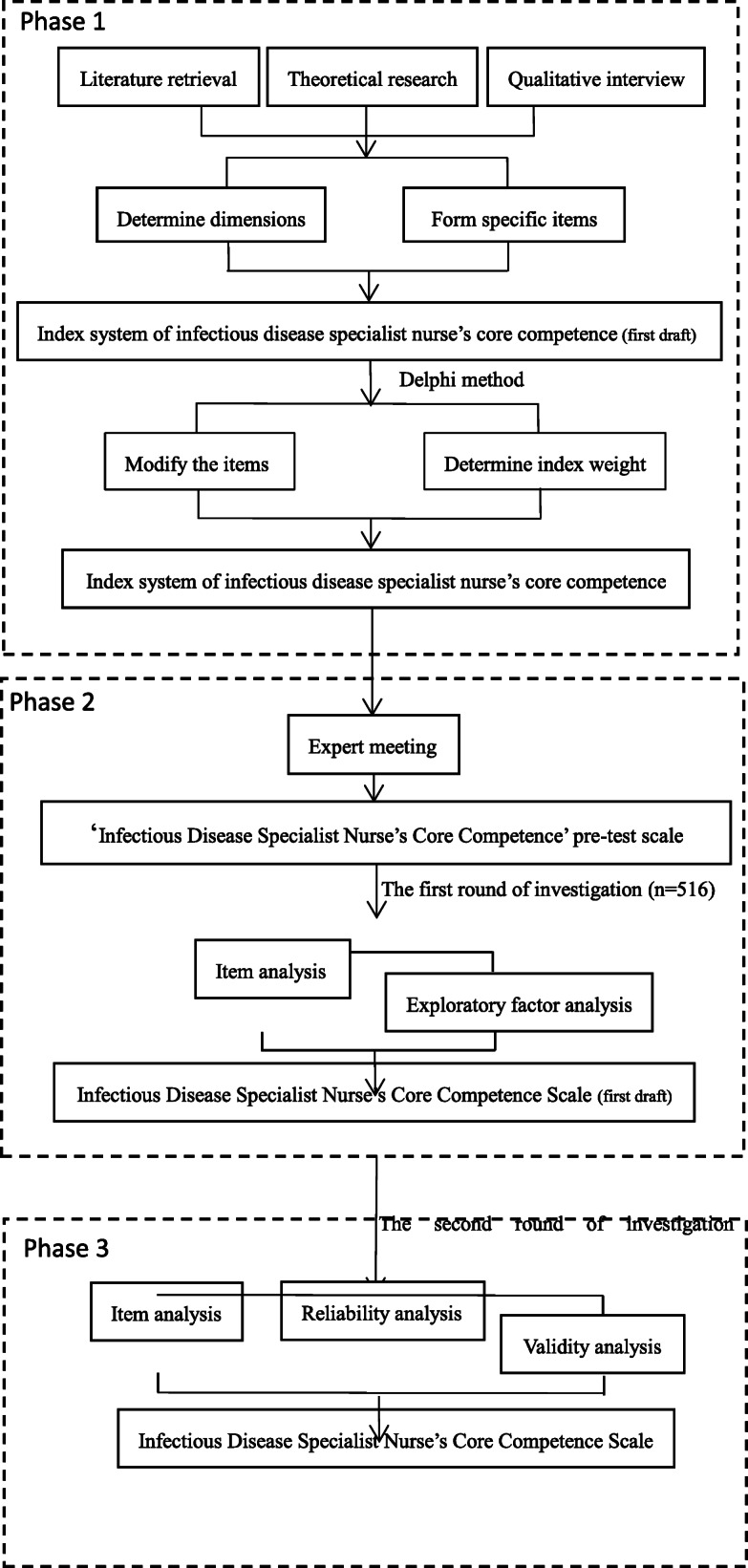
The development procedure of Infectious Disease Specialist Nurse’s Core Competence Scale

This study involved three stages as illustrated in Fig. 1.

### Phase 1: identification of dimensions and development of items

In the first stage, through literature review, theoretical analysis and qualitative interview, we constructed the first draft of the core competence evaluation index system of infectious disease specialist nurses which was composed of 6 primary indicators, 17 secondary indicators and 48 tertiary indicators, and adopted the 5-point scoring method. The primary indicators were Nursing Abilities for infectious diseases, Infection Prevention and Control Abilities, Responsiveness to Infectious Diseases, Professional Development Abilities, Communication and Management Abilities and Professionalism and Humanistic Accomplishment. Then, we invited experts in the field of specialized medical treatment and nurses of infectious diseases for Delphi consultation [16]. The inclusion criteria of consultation experts were as follows: (a) has engaged in clinical nursing or medical work of infectious diseases at least 15 years; (b) has intermediate level title or above; (c) has bachelor degree or above; (d) voluntarily participates in the research. Through Delphi expert consultation, the index system was scored and modified, and the judgment coefficient, authority coefficient and familiarity degree of Delphi experts were 0.933, 0.923 and 0.913 respectively. The core competence index system of infectious disease specialist nurses was finally established, which included 6 primary indicators, 16 secondary indicators and 47 tertiary indicators [17].

### Phase 2: preliminary evaluation and exploration of infectious disease specialist Nurse’s Core competence scale

Then, through the panel meeting, we compiled the index system into ‘Infectious Disease Specialist Nurse’s Core Competence’ pretest scale which included 47 items. Before the formal investigation, the preliminary scale was distributed to 5 head nurses to test the level of item comprehension, appropriateness of the font size, survey structure and item length. The evaluation of the pre-test scale by five head nurses had good internal consistency, which was 0.851, indicating that it can be used for formal investigation. In the first round of investigation, 40 head nurses from the infectious diseases department were invited to evaluate the core competence of 516 infectious disease specialist nurses through the pre-test scale. Discrete trend, critical ratio, correlation coefficient, Cronbach’s α coefficient and factor analysis were adopted for item analysis. Through item analysis and exploratory factor analysis, we filtered the items and drafted a preliminary scale, which was composed of 5 factors and 36 items. And the scale was basically consistent with the index system of core competence of infectious disease specialist nurses constructed in the previous study.

### Phase 3: evaluation of reliability and validity of infectious disease specialist Nurse’s Core competence scale

In the third stage, we conducted the second round of questionnaire survey. The core competence of 497 infectious disease specialist nurses was evaluated by 42 head nurses with the first draft of Infectious Disease Specialist Nurse’s Core Competence Scale. We took a series of measures including item analysis, reliability test and validity test to filter the scale items and re-explore and verify the structure of the scale. The methods of item analysis were the same as above. Reliability analysis included test-retest reliability, internal consistency and split-half reliability. Validity analysis included content validity and structure validity. In the second round of questionnaire survey, about 10% of the subjects were randomly remarked, and the questionnaire was sent out again 2 weeks later to measure the test-retest reliability. Finally, a scale with high reliability and validity was formed, including 5 dimensions and 34 items.

### Data collection procedure and quality control

Before conducting questionnaire investigation, the research team explained the research purpose and meaning to the head nurses and organized relevant training among them. In the process of evaluation, one-on-one evaluation was adopted, namely, one head nurse just evaluated one infectious disease specialist nurse at a time. The head nurses were responsible for the evaluation of core competence on all specialist nurses in the departments. When the evaluation was over, 10% of the nurses who were tested would be randomly selected and be evaluated again by senior nurses who are experienced in management in the infectious disease department. The senior nurses were also explained with the research purpose and meaning and trained as well. The same evaluation approach was adopted again. The results showed that, the correlation coefficient of head nurse and senior nurse to the certain specialist nurse’s core competence was 0.896 (*P* < 0.05). This demonstrated that the head nurse’s evaluation on the specialist nurse’s core competence was reliable, with low subjective bias.

The inclusion criteria of head nurses and senior nurses: (a) have engaged in infectious disease nursing for more than 10 years; (b) have nurse in charge title or above; (c) have good communication and expression skills; (d) voluntarily participate in research. The inclusion criteria of infectious disease specialist nurses: (a) have engaged in infectious disease nursing for more than 5 years; (b) have participated in infectious disease specialist nurse training and got the certificate.

The sample size was determined by the general rule that factor analytic procedure requires a minimum of five respondents per item, but a larger sample is desirable [18, 19]. In our study, ten respondents per item were required to ensure the accuracy of factor analysis. Therefore, during the two rounds of questionnaire investigations, head nurses of infectious diseases department were selected by convenient sampling method to evaluate the core competence of infectious disease specialist nurses.

### Statistical analysis

Data were analyzed by SPSS 23.0 and Mplus 8.3 software.

For item analysis, items were screened with the Classical Test Theory [20] which included discrete trend method, critical ratio method, correlation coefficient method, Cronbach’s α coefficient method and factor analysis method. The standard deviation of item scores represented the degree of dispersion. When SD < 0.85, it indicated that the item was not able to distinguish and was to be deleted. The total score of the scale was ranked from high to low, and the relationship between the high-score group (the first 27%) and the low-score group (the last 27%) was analyzed to judge the discrimination of the scale. It was the same to the factor loading. If the total score was less than 0.4, the item needs to be deleted. If Cronbach’s α coefficient became larger after deleting the item, it indicated that the item would lower the internal consistency of the scale and should be deleted [21].

Reliability analysis referred to the consistency of the results of repeated measurement of the same object by the same method [22, 23]. For reliability analysis, we used Cronbach’s α coefficient, split-half reliability and test-retest reliability. Cronbach’s α coefficient was used to evaluate the internal consistency reliability of the scale. The scale was divided into two parts according to the order of oddness and evenness, and the correlation between them was to calculate the split-half reliability. Two weeks later, we would conduct a test-retest on the nurses marked before, and measure the test-retest reliability.

Validity analysis referred to the analysis of the accuracy of the scale [24, 25]. For validity analysis, we conducted content validity analysis and structure analysis. The validity of the content was evaluated by Delphi experts’ scores which included the content validity index of the items (I-CVI) and content validity index of the scale (S-CVI). The structure analysis contained exploratory factor analysis and confirmatory factor analysis. Index value standard: The Kaiser-Meyer-Olkin (KMO) > 0.6, χ2/df < 3, Root mean square error approximation (RMSEA) < 0.08, Comparative fit index (CFI) > 0.90, Tucker-Lewis index (TLI) > 0.90, Standard root mean-square residual (SRMR) < 0.80 [26].

### Ethical consideration

Research was approved by the ethics committee of Tangdu Hospital of Fourth Military Military Medical University, China (Number TDLL2019-09-13). Informed consent was obtained from all participants included in the study and they could withdraw from the study at any time for any reason. Moreover, they were assured that the questionnaires would only be used for research.

## Results

### Characteristics of the participant

From January to March 2021, 540 questionnaires were distributed in the first round of survey, and 516 were effectively recovered, with an effective recovery rate of 95.56%. The average age of head nurses was (42.15 ± 5.57), and the average number of years of nursing infectious diseases was (18.75 ± 6.03). The average age of infectious disease specialist nurses was (31.02 ± 5.17), and the average number of years engaged in infectious disease nursing was (9.29 ± 4.23). From May to July 2021, the second round of questionnaire survey was conducted. A total of 517 questionnaires were distributed and 497 valid questionnaires were recovered, with an effective recovery rate of 96.13%. The average age of head nurses was (41.60 ± 4.54), and the years of nursing infectious diseases were (17.36 ± 4.76). The average age of infectious disease specialist nurses was (32.17 ± 5.77), and the number of years engaged in infectious disease nursing was (9.02 ± 3.78). Other demographic data are shown in Table 1.

**Table 1:** General demographic data. N, number

Category	Characteristics	The first round of investigation (*n* = 516)		The second round of investigation (*n* = 497)	
		N	%	N	%
Head nurse	Age (years)				
	< 40	11	27.50	12	28.57
	40–50	26	65.00	29	69.05
	> 50	3	7.50	1	2.38
	Work experience in infectious diseases (years)				
	10–20	23	57.50	25	59.52
	21–30	15	37.50	17	40.48
	> 30	2	5.00	–	–
	Title				
	Nurse in charge	29	72.50	31	73.81
	Deputy chief nurse or above	11	27.50	11	26.19
	Educational background				
	Below bachelor degree	14	35.00	13	30.95
	Bachelor degree	15	37.50	17	40.48
	Master degree or above	11	27.50	12	28.57
Infectious disease specialist nurse	Sex				
	Female	483	93.60	473	95.17
	Male	33	6.40	24	4.83
	Age (years)				
	< 30	287	55.62	247	49.70
	30–40	212	41.09	243	48.89
	> 40	17	3.29	7	1.41
	Work experience in infectious diseases (years)				
	≤10	317	61.43	297	59.76
	11–20	188	36.43	194	39.03
	> 20	11	2.14	6	1.21
	Title				
	Nurse	167	32.36	165	33.20
	Nurse in charge	347	67.25	327	65.79
	Deputy chief nurse or above	2	0.39	5	1.01
	Educational background				
	Below bachelor degree	203	39.34	176	35.41
	Bachelor degree	313	60.66	321	64.59
	Marital status				
	Unmarried	201	38.95	178	35.81
	Married	301	58.33	301	60.56
	Divorce or bereavement	14	2.71	18	3.62
	Salary (Yuan)				
	< 3000	104	20.16	117	23.54
	3000–6000	299	57.95	313	62.98
	> 6000	113	21.90	67	13.48

### Preliminary evaluation and exploration of scale

#### Item analysis

As shown in Table 2, the results of item analysis of the 516 questionnaires in the first round showed that the item analysis values of each item were up to the standard and the items were to be reserved.

**Table 2:** The item analysis for Infectious Disease Specialist Nurse’s Core Competence Scale

Item	The first round of investigation (*n* = 516)						Item	The second round of investigation (*n* = 497)					
	Discrete trend	Critical ratio	Correlation coefficient	Cronbach’s α coefficient	Factor analysis	Reserve or delete		Discrete trend	Critical ratio	Correlation coefficient	Cronbach’s α coefficient	Factor analysis	Reserve or delete
1	1.021	16.581	0.622^**^	0.979	0.628	Reserve	1	1.122	21.355	0.715^**^	0.973	0.821	Reserve
2	0.972	20.142	0.697^**^	0.979	0.705	Reserve	2	1.038	23.593	0.762^**^	0.972	0.831	Reserve
3	0.912	15.951	0.613^**^	0.979	0.632	Reserve	3	1.013	22.138	0.745^**^	0.973	0.810	Reserve
4	0.869	20.128	0.699^**^	0.979	0.712	Reserve	4	1.059	18.723	0.739^**^	0.973	0.690	Reserve
5	1.248	17.749	0.661^**^	0.979	0.655	Reserve	5	0.970	21.527	0.766^**^	0.972	0.783	Reserve
6	1.082	19.372	0.705^**^	0.979	0.714	Reserve	6	1.078	23.067	0.740^**^	0.973	0.825	Reserve
7	0.976	23.963	0.771^**^	0.979	0.784	Reserve	7	0.959	22.665	0.791^**^	0.972	0.766	Reserve
8	0.871	21.604	0.738^**^	0.979	0.748	Reserve	8	1.072	17.763	0.718^**^	0.973	0.679	Reserve
9	0.892	20.788	0.746^**^	0.979	0.756	Reserve	9	0.926	23.527	0.793^**^	0.972	0.778	Reserve
10	0.984	18.935	0.706^**^	0.979	0.712	Reserve	10	1.156	17.553	0.694^**^	0.973	0.681	Reserve
11	1.182	21.266	0.727^**^	0.979	0.747	Reserve	11	1.067	14.285	0.636^**^	0.973	0.613	Reserve
12	1.062	21.683	0.737^**^	0.979	0.755	Reserve	12	0.717	23.643	0.802^**^	0.972	0.675	Delete
13	1.034	16.520	0.643^**^	0.979	0.670	Reserve	13	0.691	25.742	0.813^**^	0.972	0.516	Delete
14	0.873	16.822	0.659^**^	0.979	0.685	Reserve	14	0.872	18.311	0.714^**^	0.973	0.805	Reserve
15	0.935	21.970	0.749^**^	0.979	0.766	Reserve	15	0.870	14.852	0.655^**^	0.973	0.805	Reserve
16	0.929	18.429	0.706^**^	0.979	0.735	Reserve	16	0.893	17.143	0.712^**^	0.973	0.785	Reserve
17	0.878	20.668	0.714^**^	0.979	0.737	Reserve	17	1.072	17.014	0.679^**^	0.973	0.783	Reserve
18	0.914	23.364	0.770^**^	0.979	0.789	Reserve	18	0.864	20.680	0.709^**^	0.973	0.764	Reserve
19	1.025	21.332	0.724^**^	0.979	0.749	Reserve	19	0.865	19.991	0.741^**^	0.973	0.767	Reserve
20	1.118	25.960	0.786^**^	0.979	0.793	Reserve	20	0.899	24.869	0.783^**^	0.972	0.731	Reserve
21	0.967	19.003	0.691^**^	0.979	0.699	Reserve	21	0.920	22.991	0.765^**^	0.972	0.686	Reserve
22	1.096	25.580	0.788^**^	0.979	0.787	Reserve	22	1.081	18.759	0.738^**^	0.973	0.676	Reserve
23	0.893	21.598	0.765^**^	0.979	0.778	Reserve	23	0.923	17.195	0.694^**^	0.973	0.634	Reserve
24	0.995	27.853	0.795^**^	0.979	0.802	Reserve	24	0.901	17.764	0.725^**^	0.973	0.624	Reserve
25	0.971	27.697	0.785^**^	0.979	0.775	Reserve	25	0.860	21.877	0.746^**^	0.973	0.647	Reserve
26	0.956	22.410	0.736^**^	0.979	0.726	Reserve	26	0.873	21.980	0.736^**^	0.973	0.593	Reserve
27	1.003	20.695	0.722^**^	0.979	0.703	Reserve	27	0.923	21.604	0.770^**^	0.972	0.590	Reserve
28	1.109	20.393	0.707^**^	0.979	0.678	Reserve	28	1.015	16.471	0.645^**^	0.973	0.645	Reserve
29	0.928	25.645	0.758^**^	0.979	0.738	Reserve	29	0.863	13.593	0.612^**^	0.973	0.711	Reserve
30	0.936	18.827	0.684^**^	0.979	0.647	Reserve	30	0.865	18.466	0.712^**^	0.973	0.745	Reserve
31	0.962	18.918	0.705^**^	0.979	0.670	Reserve	31	0.868	21.328	0.703^**^	0.973	0.742	Reserve
32	1.077	17.242	0.695^**^	0.979	0.662	Reserve	32	0.889	19.560	0.705^**^	0.973	0.716	Reserve
33	1.132	16.469	0.656^**^	0.979	0.620	Reserve	33	0.855	18.025	0.688^**^	0.973	0.706	Reserve
34	0.961	19.846	0.724^**^	0.979	0.693	Reserve	34	0.942	17.373	0.667^**^	0.973	0.528	Reserve
35	0.974	26.916	0.801^**^	0.979	0.798	Reserve	35	0.972	20.765	0.752^**^	0.972	0.574	Reserve
36	1.103	30.149	0.809^**^	0.979	0.809	Reserve	36	0.933	21.960	0.773^**^	0.972	0.507	Reserve
37	1.004	25.393	0.789^**^	0.979	0.785	Reserve							
38	1.021	27.949	0.794^**^	0.979	0.794	Reserve							
39	0.912	21.392	0.733^**^	0.979	0.723	Reserve							
40	0.865	25.105	0.738^**^	0.979	0.723	Reserve							
41	0.992	26.390	0.760^**^	0.979	0.750	Reserve							
42	0.919	26.461	0.776^**^	0.979	0.761	Reserve							
43	0.893	21.335	0.699^**^	0.979	0.703	Reserve							
44	1.049	20.527	0.698^**^	0.979	0.700	Reserve							
45	0.976	14.805	0.565^**^	0.979	0.582	Reserve							
46	0.998	19.677	0.701^**^	0.979	0.713	Reserve							
47	1.009	19.919	0.704^**^	0.979	0.715	Reserve							

Note: ^**^P < 0.01

#### Exploratory factor analysis

EFA was used to construct the core competence structure model of infectious disease specialist nurses.

The KMO value was 0.971, the Bartley Sphericity test was statistically significant (χ^2^ = 25,348.591, df = 1081, *P* < 0.005), indicating that 47 items of infectious disease specialist nurses’ core competence scale were suitable for factor analysis.

Principal Component Analysis was used to extract factors and varimax was used to rotate factors to extract components with eigenvalues higher than 1. Then, delete the highest factor load < 0.4, factor load across two or more factors and the difference < 0.2, and the number of common factor included items< 3. According to the above criteria, items 3, 7, 8, 10, 20, 23, 25, 26, 32, 36, 38 were deleted, and five common factors were extracted. The cumulative contribution of variance accounted to 72.3%.

After the exploratory factor analysis, a preliminary questionnaire of core competence of infectious disease specialist nurses was formed, including 5 factors and 36 items, which was basically consistent with the index system of core competence of infectious disease specialist nurses constructed in this study. According to the results of group discussion and professional knowledge, five factors were named, namely Professional Development Abilities, Infection Prevention and Control Abilities, Nursing Abilities for Infectious Diseases, Professionalism and Humanistic Accomplishment and Responsiveness to Infectious Diseases (as seen in Table 3).

**Table 3:** Factor matrix of Infectious Disease Specialist Nurse’s Core Competence Scale

Item	Professional Development Abilities	Infection Prevention and Control Abilities	Nursing Abilities for Infectious Diseases	Professionalism and Humanistic Accomplishment	Responsiveness to Emergency Infectious Diseases
28	0.827				
40	0.781				
29	0.748				
34	0.743				
39	0.734				
27	0.733				
42	0.731				
31	0.726				
41	0.723				
30	0.718				
33	0.701				
37	0.649				
35	0.562				
16		0.779			
13		0.765			
11		0.736			
14		0.720			
17		0.720			
19		0.715			
18		0.704			
12		0.696			
15		0.681			
1			0.694		
2			0.670		
6			0.658		
4			0.626		
9			0.542		
5			0.523		
45				0.771	
46				0.761	
47				0.711	
44				0.655	
43				0.652	
21					0.643
24					0.631
22					0.600

### Reevaluation of scale

#### Item analysis

As shown in Table 2, the results of item analysis of the 497 questionnaires in the second round showed that the standard deviation of the item 12 and 13 were less than 0.85 while other items meet the requirements. Finally, a formal scale with 34 items was formed.

#### Reliability analysis

As shown in Table 4, the scale and its five dimensions have ideal internal consistency and split-half reliability. The internal consistency of each dimension ranged from 0.692 to 0.790, and the total internal consistency was 0.806. The split-half reliability of each dimension ranged from 0.764 to 0.952, and the total split-half reliability was 0.966. In addition, after two weeks, 47 infectious disease specialist nurses were randomly selected and their core competence questionnaire was scored by 3 head nurses. The test-retest reliability of each dimension ranged from 0.696 to 0.881, and the total test-retest reliability was 0.831.

**Table 4:** Reliability coefficient of Infectious Disease Specialist Nurse’s Core Competence Scale

Dimension/Scale	Reliability coefficient		
	Cronbach’s α coefficient	Split-half reliability	Test-retest reliability
Professional Development Abilities	0.692	0.952	0.881^**^
Infection Prevention and Control Abilities	0.746	0.915	0.825^**^
Nursing Abilities for Infectious Diseases	0.764	0.856	0.696^**^
Professionalism and Humanistic Accomplishment	0.781	0.888	0.843^**^
Responsiveness to Emergency Infectious Diseases	0.790	0.764	0.866^**^
Total scale	0.806	0.966	0.831^**^

Note: ^**^P < 0.01

#### Validity analysis

##### Content validity

30 infectious disease experts from 12 hospitals in 8 different provinces and cities in China were invited to evaluate the content validity of the scale. The results showed that the I-CVI was 0.828–0.897, and S-CVI was 0.869.

#### Structure validity

##### Exploratory factor analysis

**Fig. 2:**
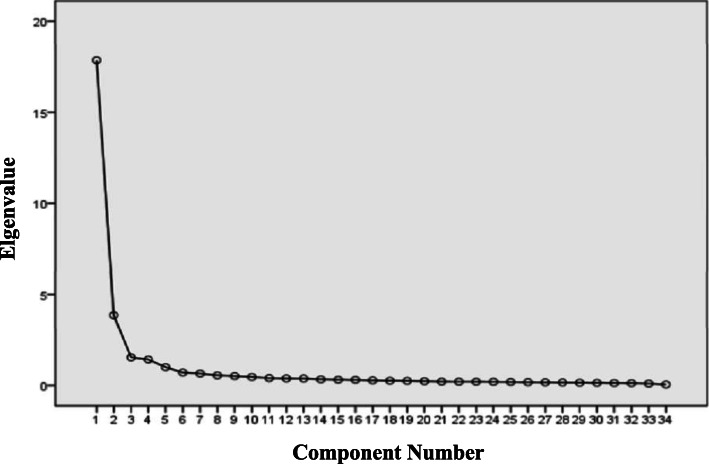
Screen plot of exploratory factor analysis for Infectious Disease Specialist Nurse’s Core Competence Scale

The results of principal component analysis of each dimension showed that among the factors of each dimension, only one had an eigenvalue greater than 1, the variance contribution rate ranged from 68.97 to 79.75%, and the load value of each dimension item was greater than 0.4, as shown in Table 5. 247 questionnaires were randomly selected from the 497 questionnaires, and exploratory factor analysis was conducted by principal component analysis. Bartlett sphericity test value was 15,650.143, KMO test value was 0.962 (*P* < 0.01). The results showed that the eigenvalues of the five factors were 17.859, 3.860, 1.543, 1.425 and 1.006 respectively, and the variance contribution rates were 52.53, 11.35, 4.54, 4.19 and 2.96% respectively. The cumulative contribution of variance rate was 75.57%. (Table 6**,** Fig. 2).

**Table 5:** Principal component analysis of each dimension of the scale

Dimension	Number of factors (eigenvalue> 1)	Variance contribution rate (%)	Item load range
Professional Development Abilities	1	71.482	0.768–0.873
Infection Prevention and Control Abilities	1	73.534	0.815–0.886
Nursing Abilities for Infectious Diseases	1	68.973	0.757–0.862
Professionalism and Humanistic Accomplishment	1	75.149	0.834–0.906
Responsiveness to Emergency Infectious Diseases	1	79.750	0.868–0.913

**Table 6:** Factor load of formal scale (34 items)

Factors and it’s items	Factor load
**Factor 1 Professional Development Abilities (eigenvalue 17.859, variance contribution rate 52.527%)**	
2 Be able to distribute, guide, supervise and manage the infectious diseases nurses	0.826
6 Be able to conduct clinical teaching	0.825
1 Be able to conduct lectures	0.823
3 Be able to train other nurses in face of emerging infectious diseases emergencies	0.813
5 Be able to collaborate with other units and departments and effectively coordinate human and material resources, etc.	0.775
9 Be able to manage materials such as drugs, consumable items, documents, instrument and equipment in infectious diseases department	0.772
7 Be able to evaluate and improve the quality of infectious diseases nursing issues and interventions	0.765
4 Be able to improve and innovate on infectious disease nursing process and protective articles	0.699
10 Be able to select and design scientific researches	0.696
8 Be able to search and retrieve literature documents by various ways and assess the quality of the literature	0.693
11 Be able to write papers	0.626
**Factor 2 Infection Prevention and Control Abilities (eigenvalue 3.860, variance contribution rate 11.354%)**	
13 Master the processes and methods of putting on the protective articles	0.807
12 Be able to correctly dispose of different medical wastes by infectious diseases patients (such as infectious diarrhea, AIDS, COVID-19, etc.)	0.806
14 Be aware of the protective requirements for different kinds of infectious diseases	0.787
15 Master the skills and processes coping with professional exposure risks such as skin mucous membrane and sharp instrument injury	0.783
17 Be aware of the requirements for different isolation techniques (isolation due to airborne transmission, contact transmission and droplet transmission)	0.769
16 Master the disinfection methods of inpatient ward and instrument and equipment in infectious diseases department	0.764
18 Master the common isolation techniques and methods	0.732
19 Master the tactics of standard and extra precautions	0.688
20 Be familiar with common physical and chemical disinfection	0.674
**Factor 3 Nursing Abilities for Infectious Diseases (eigenvalue 1.543, variance contribution rate 4.539%)**	
23 Master the emergence care skills for critical infectious diseases patients	0.655
26 Be familiar with the common diagnosis and treatment in infectious disease departments such as compression hemostasis for Sengstaken Blakemore tube, lactulose enema, and traumatic artetial blood pressure supervision paracentesis	0.643
21 Grasp the pathogenesis of common infectious diseases and the relevant knowledge of diseases including the historical epidemiology, the main nursing points and health education	0.639
23 Be familiar with the basics such as the dosage regimen, administration route, side effects and matters needing attention of drugs which are commonly taken by infectious diseases patients	0.630
25 Be able to draw up nursing plans in accordance with the different state of the different infectious diseases patients	0.598
24 Be able to deal with the symptoms and signs of common diseases such as fever, erythra, diarrhea, twitching and seizure	0.594
**Factor 4 Professionalism and Humanistic Accomplishment (eigenvalue 1.425, variance contribution rate 4.192%)**	
28 Be capable of providing psychological counseling and mental nursing	0.746
29 Be capable of providing health education for the infectious diseases patients and the public	0.742
30 Be capable of adjusting oneself and governing the stress in infectious diseases nursing work	0.717
27 Respect the patient and protect patients’ privacy and show no discrimination to the patients	0.716
31 Be passionate about infectious diseases nursing and possess the sense of professional identity of nursing infectious diseases patients	0.709
**Factor 5 Responsiveness to Emergency Infectious Diseases (eigenvalue 1.006, variance contribution rate 2.957%)**	
33 Be familiar with the response process of the infectious diseases emergencies	0.575
32 Take part in emergency drills for infectious diseases emergencies in regular terms	0.536
34 Be able to predict and recognize the infectious diseases emergencies	0.510

##### Confirmatory factor analysis

**Fig. 3:**
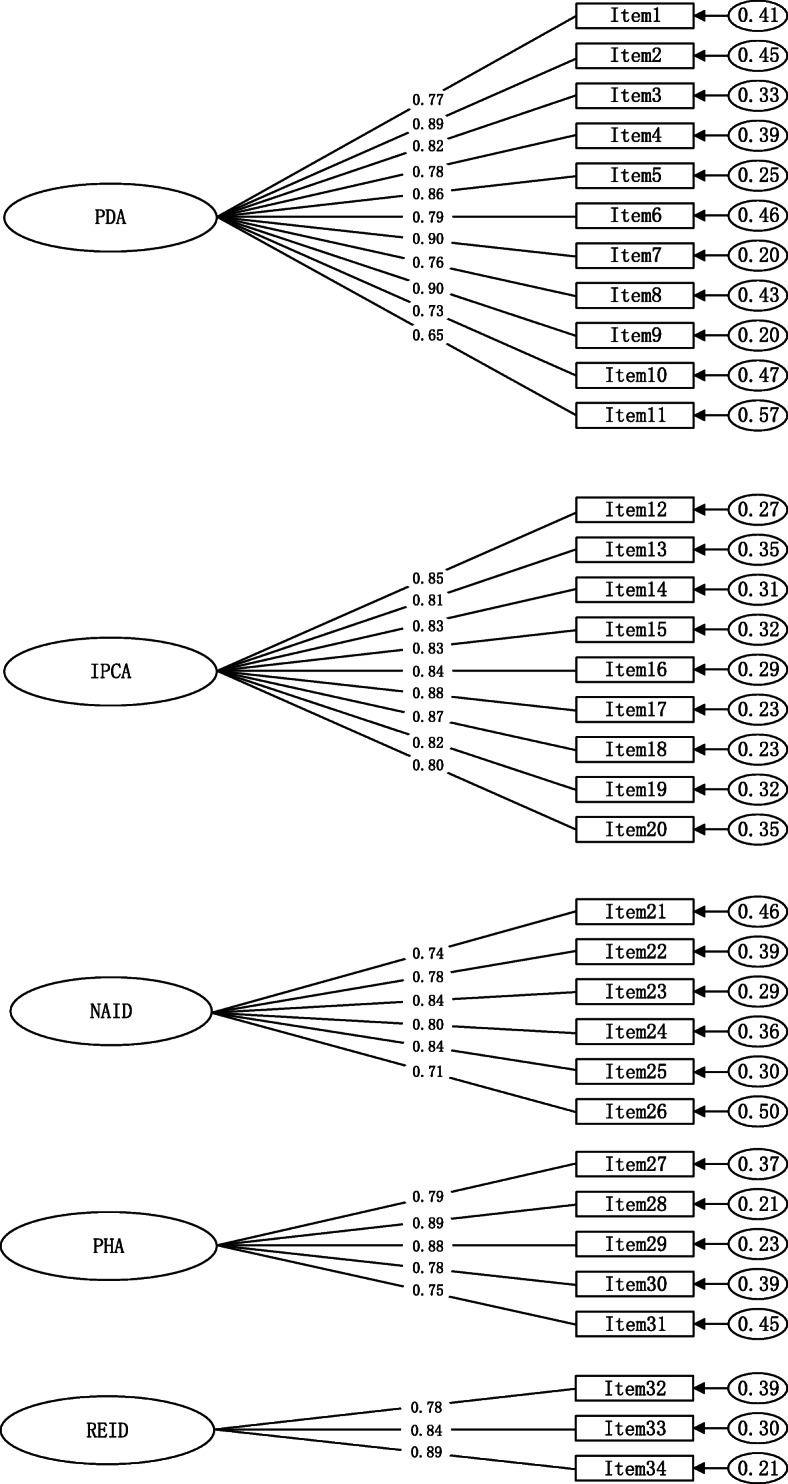
Standardized five-factor structural model of Infectious Disease Specialist Nurse’s Core Competence Scale (*n* = 250). Note: PDA = Professional Development Abilities; IPCA = Infection Prevention and Control Abilities; NAID = Nursing Abilities for Infectious Diseases; PHA = Professionalism and Humanistic Accomplishment; REID = Responsiveness to Emergency Infectious Diseases. χ^2^/df = 2.858, RMSEA = 0.062, CFI = 0.940, TLI = 0.933, SRMR = 0.051

The remaining 250 questionnaires in the second round of investigation were selected for CFA. The five-factor model was fitted by the maximum likelihood estimation method. The fitting indexes were as follows: χ^2^/df = 2.858 < 3, RMSEA = 0.062 < 0.08, CFI = 0.940 > 0.90, TLI = 0.933 > 0.90, SRMR = 0.051 < 0.8. The standard factor load model formed by confirmatory factor analysis was shown in Fig. 3. The factor load of each item was greater than 0.40, and all items had statistical significance (*P* < 0.05), indicating that the questionnaire had good structural validity.

### Discrimination analysis

Discrimination analysis was conducted on the evaluation scale of core competence of infectious disease specialist nurses. T-test and analysis of variance were used to compare the core competence and scores of 5 dimensions of infectious disease specialist nurses with different demographic characteristics. The research results are shown in Table 7. The results showed that there were significant differences in the scores of core competence and its five dimensions among infectious disease specialist nurses of different ages, working years in infectious diseases, titles, educational background, marital status and wages (all *P* < 0.05).

**Table 7:** Comparison of core competence and its dimensions of infectious disease specialist nurses with different demographic characteristics

Characteristics	Core competence	Professional Development Abilities	Infection Prevention and Control Abilities	Nursing Abilities for Infectious Diseases	Professionalism and Humanistic Accomplishment	Responsiveness to Emergency Infectious Diseases
Sex						
Female	131.78 ± 21.70	37.74 ± 9.56	38.31 ± 5.86	23.65 ± 4.17	20.76 ± 3.44	11.32 ± 2.58
Male	132.88 ± 20.46	37.79 ± 10.41	38.83 ± 5.01	24.13 ± 3.51	20.42 ± 2.74	11.71 ± 2.13
Age (years)						
< 30	120.76 ± 16.97	33.26 ± 7.58	35.99 ± 5.53	21.83 ± 3.70	19.47 ± 3.26	10.21 ± 2.36
30–40	143.68 ± 19.62^a^	42.52 ± 9.20 ^a^	40.84 ± 4.97 ^a^	25.64 ± 3.66 ^a^	22.14 ± 2.98 ^a^	12.53 ± 2.22 ^a^
> 40	111.43 ± 10.54	30.00 ± 3.46	33.86 ± 6.38	20.43 ± 3.20	17.14 ± 2.11	10.00 ± 1.82
Work experience in infectious diseases (years)						
≤ 10	124.51 ± 16.70	34.48 ± 8.05	37.00 ± 5.14	22.45 ± 3.63	19.96 ± 3.01	10.62 ± 2.28
11–20	143.56 ± 23.22 ^a^	42.84 ± 9.63 ^a^	40.56 ± 6.08 ^a^	25.63 ± 4.14 ^a^	22.02 ± 3.62 ^a^	12.50 ± 2.57 ^a^
> 20	115.33 ± 15.50	34.17 ± 6.79	32.33 ± 5.04 ^b^	20.50 ± 3.01	18.50 ± 2.07	9.83 ± 1.94
Title						
Nurse	122.62 ± 24.29	34.81 ± 10.36	35.84 ± 6.38	22.05 ± 4.58	19.53 ± 3.83	10.41 ± 2.76
Nurse in charge	136.67 ± 18.58 ^a^	39.32 ± 8.85 ^a^	39.62 ± 5.09 ^a^	24.51 ± 3.67 ^a^	21.39 ± 3.00 ^a^	11.83 ± 2.33 ^a^
Deputy chief nurse or above	119.20 ± 8.07	31.60 ± 3.28	36.40 ± 3.97	22.20 ± 1.64	18.60 ± 1.67	10.40 ± 1.51
Educational background						
Below bachelor degree	112.34 ± 15.43	30.38 ± 6.74	33.78 ± 5.41	20.37 ± 3.45	18.44 ± 3.25	9.37 ± 2.09
Bachelor degree	142.52 ± 16.47 ^a^	41.78 ± 8.45 ^a^	40.83 ± 4.34 ^a^	25.48 ± 3.29 ^a^	22.01 ± 2.77 ^a^	12.42 ± 2.11 ^a^
Marital status						
Unmarried	121.12 ± 15.80	33.25 ± 7.60	36.17 ± 5.56	21.98 ± 3.46	19.46 ± 3.14	10.25 ± 2.28
Married	139.11 ± 21.80 ^a^	40.78 ± 9.56 ^a^	39.82 ± 5.53 ^a^	24.81 ± 4.13 ^a^	21.63 ± 3.29 ^a^	12.07 ± 2.50 ^a^
Divorce or bereavement	116.17 ± 16.28	31.39 ± 7.24	34.94 ± 5.04	21.28 ± 3.93	18.67 ± 3.14	9.89 ± 1.74
Salary (Yuan)						
< 3000	113.79 ± 21.35	31.66 ± 8.62	33.30 ± 6.34	20.66 ± 4.03	18.47 ± 3.74	9.71 ± 2.59
3000–6000	138.81 ± 17.64 ^a^	40.08 ± 9.08 ^a^	40.18 ± 4.40 ^a^	24.87 ± 3.55 ^a^	21.63 ± 2.89 ^a^	12.05 ± 2.20 ^a^
> 6000	130.75 ± 20.84 ^b^	37.46 ± 8.90 ^b^	38.52 ± 5.55 ^b^	23.31 ± 4.22 ^b^	20.58 ± 3.18 ^b^	10.87 ± 2.69 ^b^

*Abbreviation*: ^a^: Comparison of the first and second items (*P* < 0.05)

^b^: Comparison of the first and third items (*P* < 0.05)

## Discussion

### The significance and innovation of the scale

Among all present researches, there was no tool targeted at evaluating the infectious disease specialist nurse’s core competence. This study is of great significance to some degree since this study aims at establishing an effective system targeted at evaluating the infectious disease specialist nurse’s core competence, which can provide reference to the training and assessment of the infectious disease specialist nurses and improve the quality of infectious disease nursing care. And the study is innovative because the Infectious Disease Specialist Nurse’s Core Competence Scale compiled by the research team under the theoretical guidance framework of the Core Competence Evaluation Index System of Infectious Disease Nurses constructed in the early stage fills the gap in the field of the core competence evaluation of the infectious disease specialist nurses.

### The practicability of the scale

The scale was under the guidance of Core Competence Theory [27] and designed in combination with the characteristics of the infectious diseases (Ma & Cao, 2018; Wu et al., 2020) and the actual situation of the infectious diseases [28]. In the process of constructing the scale, every dimension was endowed with a mission for infectious disease specialist nurses to fulfill. And the missions required the nurses to be able to give lectures, do scientific researches and undertake administrative work so as to advance professional development; to be able to strictly implement infection prevention and control; to be armed with solid theoretical knowledge and operational skills so as to undertake the nursing work of the infectious disease patients; to able to respect the infectious disease patients and to be able to respond to Emergency Infectious Diseases. The scale was designed for scientifically and effectively evaluating the core competence of the infectious disease nurses and providing measurement tool for carrying out clinical infectious disease nursing care and improving the specialist nurses’ abilities. The higher the score of the scale the nurse got, the higher the level of the nurse’s core competence was. In this way, the scale was of practicability.

### The scientificity of the scale

The Infectious Disease Specialist Nurse’s Core Competence Scale, featured as high reliability and validity, was finally established with 5 factors and 34 items after preliminary evaluation and exploration in the first round and re-evaluation in the second round. The cumulative explanatory variance of the five factors was 75.569%, indicating that the five factors could explain the difference in core competence of infectious diseases specialist nurses to the extent of 75.569%. The scale had a clear structure and was roughly consistent with the index system constructed in the first part, which confirmed the rationality of the design structure. Through a series of methods such as a large number of literature review, theoretical analysis, qualitative interviews, expert correspondence, etc., the comprehensive consideration of the core competence of infectious disease specialist nurses was transformed into an evaluation tool, which had a certain degree of scientificity.

On the basis of reliability and validity evaluation, the differences of core competence and its dimension scores of infectious disease specialist nurses in different demographic characteristics were compared, and the influencing factors of core competence were preliminarily explored. The core competence of infectious disease specialist nurses aged 31–40 was higher than that of low and high age group; the core competence of specialist nurses who have worked in infectious disease nursing for 11–20 years were higher than those who have worked for less than 10 years and more than 20 years; the core competence of nurse in charge was higher than that of the nurse or the deputy chief nurse or above; specialist nurses with middle salary level had the highest score in core competence. The nurse in charge has longer working years and accumulated a lot of clinical experience in infectious disease nursing than younger nurse. At the same time, compared with older nurses, the nurse in charge was the core backbone of the Department and was mainly responsible for the nursing and management of infectious diseases, so the level of core competence was the highest. The core competence score of nurses with bachelor degree was higher, because they have received a higher education level and can better master clinical skills and improve clinical nursing ability [29]. Zuriguel-p é rez et al. [30] found that highly educated nurses often have better critical thinking in nursing work, which is also an important aspect of core competence. The core competence of married specialist nurses was higher than that of unmarried or divorced nurses, which may be related to the family support from relatives for nurses’ work. Especially the nurses in the Department of infectious diseases are facing great work pressure and the risk of occupational exposure [31]. Family support and recognition are the driving force of their work, which can make them better put into work [32, 33].

### Limitations and perspectives

In this study, cross group measurement invariance analysis on the scale was not done. So it was not clear whether there were differences in the application in different groups with different characteristic [34]. And In the next step, we will analyze the invariance of cross group measurement to figure out the differences in the application in different groups. Besides, we will introduce Generalizability Theory (GT) and apply it in the re-evaluation of the scale so that we can further verify and improve the reliability and validity of the scale [35].

## Conclusions

This study is the first one to develop and validate a scale for measuring the core competence of infectious disease specialist nurse. The scale in this study comprises 34 items, 11 items in “Professional Development Abilities”, 9 items in “Infection Prevention and Control Abilities”, 6 items in “Nursing Abilities for Infectious Diseases”, 5 items in “Professionalism and Humanistic Accomplishment”, and 3 items in “Responsiveness to Emergency Infectious Diseases”. The scale’s validity and reliability for measuring infectious disease specialist nurses’ core competence were confirmed.

## Data Availability

The datasets generated and analyzed during the current study are not publicly available due to the protection of the privacy of consulting experts but are available from the corresponding author (906963251@qq.com) on reasonable request.
